# Management of myelofibrosis after ruxolitinib failure

**DOI:** 10.1007/s00277-020-04002-9

**Published:** 2020-03-20

**Authors:** Claire N Harrison, Nicolaas Schaap, Ruben A Mesa

**Affiliations:** 1 Guy’s and St Thomas’ Hospital Foundation Trust, Westminster Bridge Rd, London, SE1 7EH UK; 2 Radboud University Medical Centre, Nijmegen, Netherlands; 3 University of Texas Health Science Center at San Antonio, San Antonio, TX USA

**Keywords:** Myelofibrosis, Ruxolitinib, Fedratinib, Momelotinib, Pacritinib

## Abstract

Myelofibrosis is a BCR-ABL1–negative myeloproliferative neoplasm characterized by anemia, progressive splenomegaly, extramedullary hematopoiesis, bone marrow fibrosis, constitutional symptoms, leukemic progression, and shortened survival. Constitutive activation of the Janus kinase/signal transducers and activators of transcription (JAK-STAT) pathway, and other cellular pathways downstream, leads to myeloproliferation, proinflammatory cytokine expression, and bone marrow remodeling. Transplant is the only curative option for myelofibrosis, but high rates of morbidity and mortality limit eligibility. Several prognostic models have been developed to facilitate treatment decisions. Until the recent approval of fedratinib, a JAK2 inhibitor, ruxolitinib was the only available JAK inhibitor for treatment of intermediate- or high-risk myelofibrosis. Ruxolitinib reduces splenomegaly to some degree in almost all treated patients; however, many patients cannot tolerate ruxolitinib due to dose-dependent drug-related cytopenias, and even patients with a good initial response often develop resistance to ruxolitinib after 2–3 years of therapy. Currently, there is no consensus definition of ruxolitinib *failure*. Until fedratinib approval, strategies to overcome ruxolitinib resistance or intolerance were mainly different approaches to continued ruxolitinib therapy, including dosing modifications and ruxolitinib rechallenge. Fedratinib and two other JAK2 inhibitors in later stages of clinical development, pacritinib and momelotinib, have been shown to induce clinical responses and improve symptoms in patients previously treated with ruxolitinib. Fedratinib induces robust spleen responses, and pacritinib and momelotinib may have preferential activity in patients with severe cytopenias. Reviewed here are strategies to ameliorate ruxolitinib resistance or intolerance, and outcomes of clinical trials in patients with myelofibrosis receiving second-line JAK inhibitors after ruxolitinib treatment.

## Introduction

Myelofibrosis (MF) is a BCR-ABL1–negative myeloproliferative neoplasm (MPN) characterized by bone marrow fibrosis, anemia, progressive splenomegaly, extramedullary hematopoiesis, debilitating constitutional symptoms, cachexia, leukemic progression, shortened survival, and compromised quality of life (QoL) [1, 2]. MF may be de novo (primary MF) or secondary to polycythemia vera (PV) or essential thrombocythemia (ET). Approximately 90% of patients with MF carry mutations in any of 3 driver genes: Janus kinase 2 (*JAK2*) in ~ 60% of cases, calreticulin (*CALR*) in ~ 20%, and myeloproliferative leukemia virus oncogene (*MPL*) in ~ 10% [3, 4]. Mutant proteins activate the Janus kinase/signal transducers and activators of transcription (JAK-STAT) pathway and other pathways downstream, leading to myeloproliferation, proinflammatory cytokine expression, and bone marrow remodeling [5, 6]. Additionally, “subclonal” mutations in certain genes, including *LNK*, *CBL*, *TET2*, *ASXL1*, *IDH1*, *IDH2*, *EZH2*, *DNMT3A*, *SF3B1*, *TP53*, *U2AF1*, and *SRSF2*, are thought to contribute to disease progression and leukemic transformation [1, 4].

Because MF is associated with a heterogenous clinical phenotype, stratifying patients by prognosis can facilitate choice of appropriate treatment and identify candidates for high-risk procedures such as transplant [7]. The International Prognostic Scoring System (IPSS), used at diagnosis, utilizes five independent predictors of inferior survival to determine disease risk in primary MF: age > 65 years, hemoglobin (Hgb) < 10 g/dL, white cell count > 25 × 10^9^/L, circulating blasts ≥ 1%, and presence of constitutional symptoms [8]. The presence of 0, 1, 2, or ≥ 3 adverse features indicates low-, intermediate 1-, intermediate 2-, or high-risk disease, respectively, and corresponding median survival times range from approximately 11.3 to 2.3 years [8]. The Dynamic IPSS (DIPSS) can be used to stratify prognosis at any time during the disease course [9]. The DIPSS includes the same five prognostic factors as the IPSS but ascribes greater weight to low Hgb (2 points instead of 1); risk scoring is modified accordingly, and corresponding median survival estimates for low-, intermediate 1-, intermediate 2-, and high-risk diseases range from not reached to 1.5 years [9]. The subsequent DIPSS-Plus includes three additional independent prognostic factors: red blood cell (RBC) transfusion dependence, platelet count < 100 × 10^9^/L, and unfavorable karyotype [10]. The presence of DIPSS-Plus–defined low-, intermediate 1-, intermediate 2-, or high-risk disease is associated with corresponding median survivals of approximately 15.4 years, 6.5 years, 2.9 years, and 1.3 years, respectively [10].

Transplant is the only curative option for MF, but a high rate of transplant-related morbidity and mortality in a fragile and typically older patient population underscores the need for reliable prognostic models that can guide risk-benefit decisions in transplant-eligible patients (generally considered to be aged 70 years or younger) [1, 11–14]. To this end, more recently developed prognostic models supplement (or replace) morphologic and clinical metrics in the IPSS and DIPSS with assessment of prognostically relevant molecular mutations and cytogenetic abnormalities. The mutation-enhanced IPSS (MIPSS70) integrates clinical data with molecular information and bone marrow fibrosis grade in a prognostic model aimed to facilitate treatment decisions for transplantation-aged patients (aged ≤ 70 years) [14]. The MIPSS70 incorporates six clinical risk variables (Hgb < 10 g/dL, leukocytes > 25 × 10^9^/L, platelets < 100 × 10^9^/L, circulating blasts ≥ 2%, bone marrow fibrosis grade ≥ 2, and constitutional symptoms), five identified high-molecular risk (HMR) mutations (*ASXL1*, *SRSF2*, *EZH2*, *IDH1*, and *IDH2* [4]), and one favorable mutation (*CALR* type 1/like [15]), in patients with pre-fibrotic MF or overt primary MF [14]. The MIPSS70 defined three risk categories (low, intermediate, and high), with predicted 5-year overall survival (OS) ranging from 95 to 29% [14]. An extension of the MIPSS70, the MIPSS70+, incorporates cytogenetic risk (favorable vs. unfavorable) into the prognostic model and considers the same HMR mutations but only three clinical risk factors (Hgb < 10 g/dL, circulating blasts ≥ 2%, and constitutional symptoms) [14]. The MIPSS70+ delineates four risk categories (low, intermediate, high, and very high), with 5-year OS ranging from 91 to 7% [14]. The subsequent MIPSS70+ (version 2.0) further stratifies the cytogenetic risk category to very high risk (VHR), unfavorable, and favorable; incorporates *U2AF1Q157* as an additional HMR mutation; and also includes sex- and severity-adjusted prognostically discriminative Hgb thresholds (severe anemia, defined as Hgb concentrations of < 8 g/dL in women and of < 9 g/dL in men, and moderate anemia, defined as Hgb of 8 g/dL to 9.9 g/dL in women and of 9 g/dL to 10.9 g/dL in men) [16].

The genetically inspired IPSS (GIPSS) is a prognostic model based solely on molecular mutations and karyotype in patients with MF [17]. The GIPSS considers the prognostic relevance of driver mutations (e.g., presence of *CALR* type 1/like mutations) and of type and number of HMR mutations [17]. Among 641 patients with primary MF, multivariable analysis identified VHR karyotype, unfavorable karyotype, absence of type 1/like *CALR* mutation, and presence of *ASXL1*, *SRSF2*, or *U2AF1Q157* mutations, as independent predictors of poor survival [17]. The GIPSS defined four prognostic risk categories (low, intermediate 1, intermediate 2, and high), with 5-year OS ranging from 94 to 14% [17].

Finally, the MF transplant scoring system (MTSS) was created to predict post-transplant outcomes for patients with primary or secondary (post-ET or post-PV) MF, based on clinical, molecular, and transplant-specific information [18]. The MTSS identified age ≥ 57 years, Karnofsky performance status < 90%, platelet count < 150 × 10^9^/L, leukocyte count > 25 × 10^9^/L before transplantation, HLA-mismatched unrelated donor, *ASXL1* mutation, and non-*CALR*/*MPL* driver mutation genotype, as independent predictors of survival. The four MTSS risk categories (low, intermediate, high, and very high) predict 5-year OS rates post-transplant ranging from 83 to 22% [18].

**Fig. 1:**
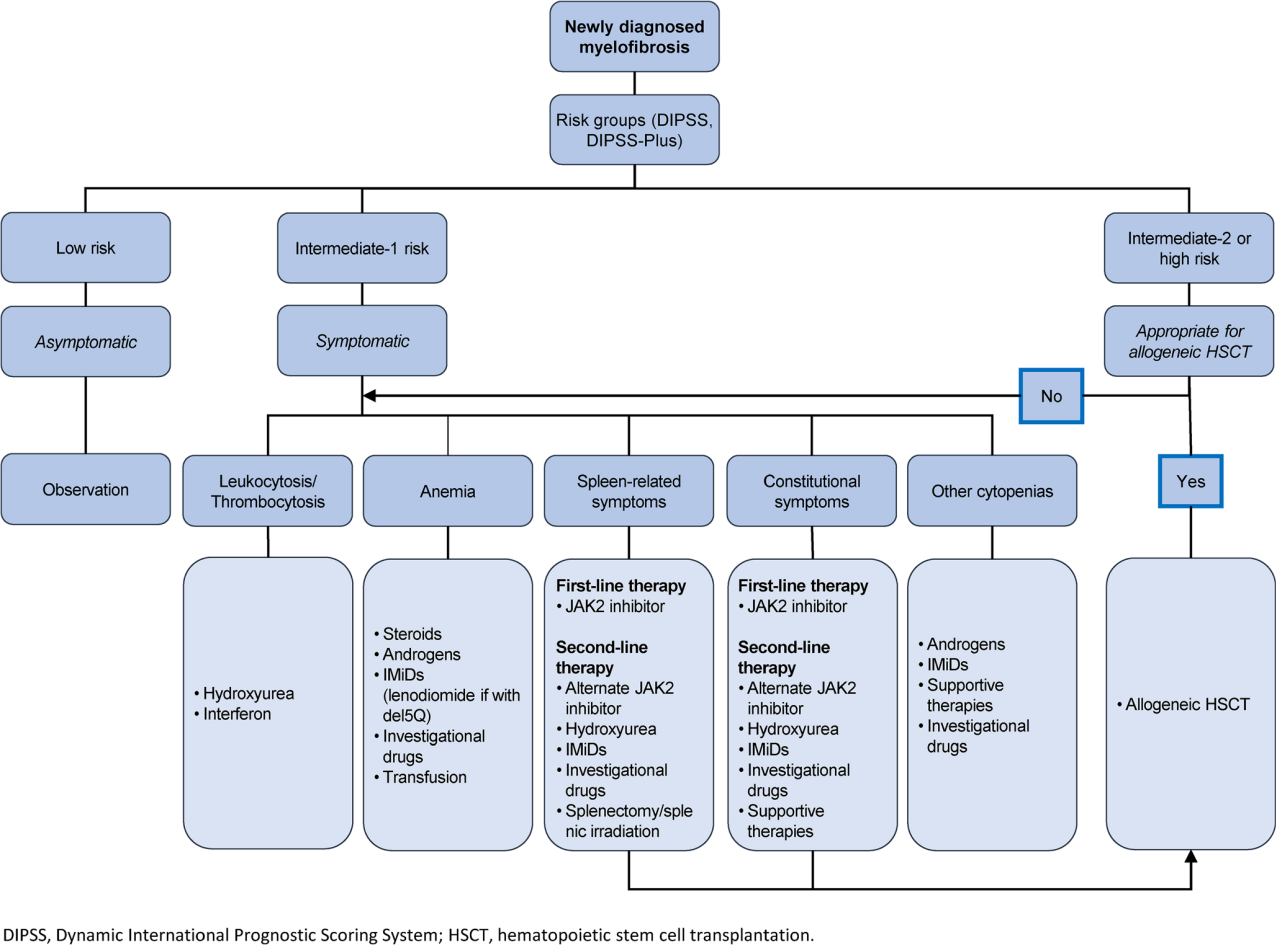
Proposed treatment algorithm for primary myelofibrosis [19]

For those who do not undergo transplant, treatment remains palliative, targeted at clinical aspects of the disease in need of treatment, such as cytopenias, splenomegaly, and constitutional symptoms (Fig. 1). Asymptomatic patients with low/intermediate 1-risk MF may not require any therapy. Androgens, prednisone, danazol, thalidomide, and lenalidomide have been used to treat MF-related anemia, and hydroxyurea, JAK2 inhibitors, and other agents have been used to treat splenomegaly [1, 20]. No MF drug therapy has yet clearly been proven to be disease modifying. For the majority of patients with MF, goals of drug therapy include reducing symptoms, decreasing risk of leukemic transformation, prolonging survival, and improving QoL.

JAK2 inhibitors reduce JAK2 and STAT phosphorylation resulting in reduced cellular proliferation and induction of apoptosis [21]. Between 2011 and 2019, ruxolitinib, a JAK1/JAK2 inhibitor, was the only approved drug treatment option for patients with intermediate- or high-risk MF [22]. Most MF patients achieve at least some degree of spleen size reduction with ruxolitinib [23–26]. However, even patients with a good initial response may lose response to ruxolitinib after 2–3 years of therapy [25–28]. In the phase III COMFORT-I [24] and COMFORT-II [23] clinical trials, approximately one half of patients discontinued ruxolitinib within 3 years and three fourths did so by 5 years [25, 26]. In clinical practice, ruxolitinib discontinuation rates can range from ~ 40 to 70% during the first year of treatment but are highly variable [28, 29]. Median survival after ruxolitinib discontinuation is generally poor, ranging from ~ 6 months to 2 years [27, 28, 30].

Described below are current concepts related to ruxolitinib failure and attempts to overcome it, and outcomes of second-line JAK inhibitor therapy in the post-ruxolitinib setting, with a focus on fedratinib, currently the only approved JAK inhibitor indicated for treatment of patients with MF previously treated with ruxolitinib.

## Ruxolitinib failure

Until recently, patients who were relapsed or refractory to ruxolitinib, or who could not tolerate the drug, had no other approved treatment options, and strategies to overcome ruxolitinib failure were mainly different approaches to continued ruxolitinib therapy, including ruxolitinib dosing modifications and ruxolitinib rechallenge after a period of dosing interruption. Furthermore, some data suggest that transplantation in the setting of ruxolitinib failure (primary failure or loss of response) is associated with worse outcomes [31]. However, there is now an alternative option for patients who do not respond, lose response, or cannot tolerate ruxolitinib. In August 2019, the United States Food and Drug Administration (FDA) approved fedratinib (INREBIC^®^), an oral selective inhibitor of JAK2, for treatment of adult patients with intermediate 2- or high-risk primary or secondary (post-PV or post-ET) MF, including patients previously treated with ruxolitinib [32].

There is currently no consensus definition of ruxolitinib failure. What has been described as the “heterogeneity of treatment failure” [33] can include primary resistance (which fortunately seems to be rare), loss of an initial response, intolerance to the drug, or progressive disease during treatment, all of which may be linked to the ruxolitinib dose [20, 34–36]. Studies of second-line JAK inhibitor therapy in patients previously exposed to ruxolitinib have used varying definitions of ruxolitinib failure for clinical trial eligibility (Table 1). As described in further detail below, ruxolitinib resistance or intolerance in the single-arm phase II JAKARTA2 study of fedratinib in patients with intermediate- or high-risk MF previously treated with ruxolitinib (NCT01523171) was based on investigator judgment after what could have been very limited prior ruxolitinib exposure [34]. No information was reported on the extent of prior ruxolitinib (or other JAK inhibitors) exposure or outcomes of ruxolitinib therapy in the PERSIST-2 study of pacritinib, a JAK2/FLT3 inhibitor, versus best available therapy (BAT) [36], and eligibility criteria in the phase III SIMPLIFY-2 study of momelotinib, a dual JAK1/JAK2 inhibitor, versus BAT-limited enrollment to patients with MF who experienced toxicity during prior or current ruxolitinib therapy [35]. So far, it remains unclear whether the reason for discontinuing ruxolitinib may influence the outcome of subsequent MF treatment, but this could be an important aspect influencing patient prognosis and choice of subsequent therapy [20].

**Table 1:** Ruxolitinib-related eligibility criteria for large clinical trials of fedratinib, pacritinib, and momelotinib in patients previously treated with ruxolitinib [34–37]

Trial	Treatment(s)	Eligibility criteria related to prior (or current) ruxolitinib exposure
JAKARTA2 (original analysis)	Fedratinib 400 mg QD	All patients previously received ruxolitinib (intention-to-treat (ITT) cohort; *N* = 97). Patients were classified as ruxolitinib resistant or ruxolitinib intolerant per investigator discretion • Resistant: ruxolitinib treatment for ≥ 14 days with lack of response, evidence of disease progression, or loss of response • Intolerant: discontinued ruxolitinib due to unacceptable toxicity after any duration of therapy
JAKARTA2 (updated analysis)	Fedratinib 400 mg QD	Met new stringent criteria for ruxolitinib relapsed, refractory, or intolerant (Stringent Criteria Cohort, *n* = 79) • Relapsed: ruxolitinib treatment for ≥ 3 months with spleen regrowth (< 10% spleen volume reduction or < 30% spleen size decrease from baseline), following an initial response. Response to ruxolitinib was defined as ≥ 50% reduction in spleen size for baseline spleen size > 10 cm from the LCM (or ≥ 35% reduction from baseline spleen volume), a non-palpable spleen for baseline spleen size between 5 and 10 cm from the LCM, or not eligible for spleen response for baseline spleen < 5 cm from the LCM • Refractory: ruxolitinib treatment for ≥ 3 months with < 10% spleen volume reduction or < 30% decrease in spleen size from baseline • Intolerant: ruxolitinib treatment for ≥ 28 days complicated by development of red blood cell transfusion requirement (≥ 2 units per month for ≥ 2 months), or grade ≥ 3 thrombocytopenia, anemia, hematoma, and/or hemorrhage
PERSIST-2	Pacritinib 200 mg BID Pacritinib 400 mg QD BAT	Prior treatment with 1 or 2 other JAK inhibitors was allowed. 95 of 221 patients enrolled were previously exposed to ruxolitinib (pacritinib 200 mg BID *n* = 31; pacritinib 400 mg QD n = 31; BAT *n* = 33)
SIMIPLIFY-2	Momelotinib 200 mg QD BAT	Current (i.e., ongoing at entry) or previous treatment with ruxolitinib for ≥ 28 days and either required RBC transfusion while on ruxolitinib or required a dose adjustment of ruxolitinib to < 20 mg BID and also had anemia, grade 3 thrombocytopenia, or grade ≥ 3 bleeding event during ruxolitinib treatment

*BAT* best available therapy, *LCM* left costal margin, *RBC* red blood cell

### Primary resistance

Most patients experience some degree of spleen reduction with ruxolitinib, and primary resistance is very uncommon (2–5%) [21, 23–26]. Inadequate response to ruxolitinib has been defined as lack of target reduction in spleen size and/or persistence of constitutional symptoms during therapy [38]. In accordance with the International Working Group-Myeloproliferative Neoplasms Research and Treatment (IWG-MRT) and European LeukemiaNet (ELN) consensus guidelines [2], a ≥ 35% reduction in spleen volume detected on MRI or CT scan has been used as the primary endpoint in MF clinical trials of JAK inhibitors [34–36, 39–41]. However, a minimum degree of splenic volume or size reduction that confers a therapeutic benefit to patients has not been established and may be individual for each patient [20]. In a landmark analysis at 24 weeks across both COMFORT studies, patients who had spleen volume reductions of ≥ 10% during ruxolitinib therapy had better prognosis than those who did not [42]. However, the hazard ratios for deeper spleen responses (e.g., ≥ 25% volume reduction) had overlapping confidence intervals, failing to demonstrate that increasing spleen volume or spleen length reductions significantly improved OS [42].

It has been suggested that MF that is primary refractory to ruxolitinib is indicated by the absence of onset of any clinical response within 28 days of starting treatment [20]. The prescribing information for ruxolitinib recommends a longer timeframe, due to the need to establish an effective dose and, potentially, to overcome side effects such as headache and dizziness that can occur early in therapy [22]. Responses are typically seen within the first 6 months of ruxolitinib treatment, and consideration should be given to discontinuing ruxolitinib if no spleen or symptom response is observed within that time [22, 43, 44]. A suboptimal response to ruxolitinib might be indicated by either the failure to achieve a minimum of clinical improvement (CI; i.e., achievement of anemia, spleen, or symptom response, without progressive disease or increase in severity of anemia, thrombocytopenia, or neutropenia [2]) within 12 weeks of starting treatment or a “mixed response” wherein supervening adverse events (e.g., cytopenias) complicate CI or better clinical response, particularly if ruxolitinib dose reduction or treatment interruption is necessary [20].

Potential predictors of ruxolitinib resistance have been investigated. In a study of 408 patients with primary or secondary MF, patients receiving ruxolitinib were significantly less likely to have a spleen response if they had pronounced splenomegaly (≥ 10 cm below the costal margin), a ≥ 2-year time interval between MF diagnosis and initiation of ruxolitinib treatment, or were transfusion dependent when starting treatment [45]. In another study, samples from 95 patients with MF treated with ruxolitinib were subject to next-generation sequencing (NGS) analysis; patients with 3 or more mutations at baseline had a reduced likelihood of achieving a spleen response during therapy and were more likely to lose a response, i.e., had a shorter time to treatment failure [46].

Ruxolitinib resistance may be ameliorated by increasing dose (if tolerable), and implementing a gradual dose escalation scheme might mitigate the potential for worsening anemia during early therapy [47]. Resistance might also be overcome when combining ruxolitinib with another agent, although combination regimens with ruxolitinib in patients with MF have thus far been generally disappointing [48]. In some instances, ruxolitinib rechallenge can induce new spleen responses (described below).

### Relapse/loss of response to ruxolitinib

Response criteria in the IWG-MRT/ELN consensus guidelines define relapse as no longer meeting criteria for at least CI after having achieved a complete or partial response or CI, or loss of anemia or spleen responses persisting for at least 1 month [2]. Secondary resistance to ruxolitinib is not uncommon; of patients enrolled in the COMFORT-I and COMFORT-II studies who discontinued ruxolitinib therapy by 3 years, most did so primarily because of loss of response and/or disease progression [49, 50]. Criteria for progressive disease, with regard to spleen size, varied between the COMFORT-I and COMFORT-II studies; these criteria were a ≥ 25% increase in spleen volume from *baseline* in the COMFORT-I study and a ≥ 25% increase in spleen volume from *nadir* in the COMFORT-II study [23, 24, 42].

Loss of a previously confirmed clinical response to ruxolitinib is typically observed as some degree of spleen regrowth, but may include resumption or exacerbation of constitutional symptoms, or disease progression [20, 34, 38]. Disease progression may present in the context of worsening leukocytosis, thrombocytopenia, or anemia, or an increase in circulating blasts [2, 20]. Late-onset cytopenias developing after 6–12 months of treatment at a stable ruxolitinib dose may also reflect disease progression [20]. Progression to AML is defined as a persistent blast count in the bone marrow or peripheral blood of ≥ 20%, but blast percentage increases of < 20% are also important [2].

Chronic exposure to JAK inhibitors has been shown to lead to a loss of response in vitro, in animal models, and in patients with MF [51]. Secondary mutations in the JAK2 kinase domain have not been identified in JAK inhibitor–resistant patients, suggesting mutation-independent mechanisms may mediate survival of MPN cells in the setting of chronic JAK inhibition [51]. In vitro data suggest heterodimerization of JAK2 with other JAKs (JAK1/TYK2) may reactivate JAK-STAT signaling in the presence of chronic JAK2 inhibition with ruxolitinib [52]. However, none of these purported biological mechanisms of resistance have been demonstrated in patients to date.

### Ruxolitinib intolerance

Cytopenias are a hallmark of MF, and dose-dependent treatment-related cytopenias are an expected side effect of drugs that target JAK/STAT signaling, which is essential to normal hematopoiesis [21, 53]. Hematologic adverse events are a leading cause of ruxolitinib discontinuation, and a majority of patients in ruxolitinib clinical trials and observational studies have had dose reductions or interruptions due to development or exacerbation of cytopenias [26, 28, 49, 54–58]. In the COMFORT-I study, grade 3–4 anemia was reported for 45% of patients in the initial 24-week treatment period [24]. In the COMFORT-II study, ruxolitinib dose modifications due to thrombocytopenia during the initial 48-week treatment period were reported for 41% of patients in the ruxolitinib arm and 1% in the BAT arm [23]. In both COMFORT studies, however, these cytopenias were not common reasons for drug discontinuation. Anemia and thrombocytopenia tend to occur during early ruxolitinib therapy and generally do not appear to increase in severity with longer-term treatment [49]. Ruxolitinib 5 mg BID dosing is recommended for patients with MF with platelet counts of 50 × 10^9^/L to < 100 × 10^9^/L [22]. However, long-term maintenance at a 5 mg BID dose has shown limited responses in some studies, and continued treatment at this dose should be limited to patients for whom the benefits outweigh the potential risks [22, 59, 60].

### Ruxolitinib discontinuation/withdrawal

**Fig. 2:**
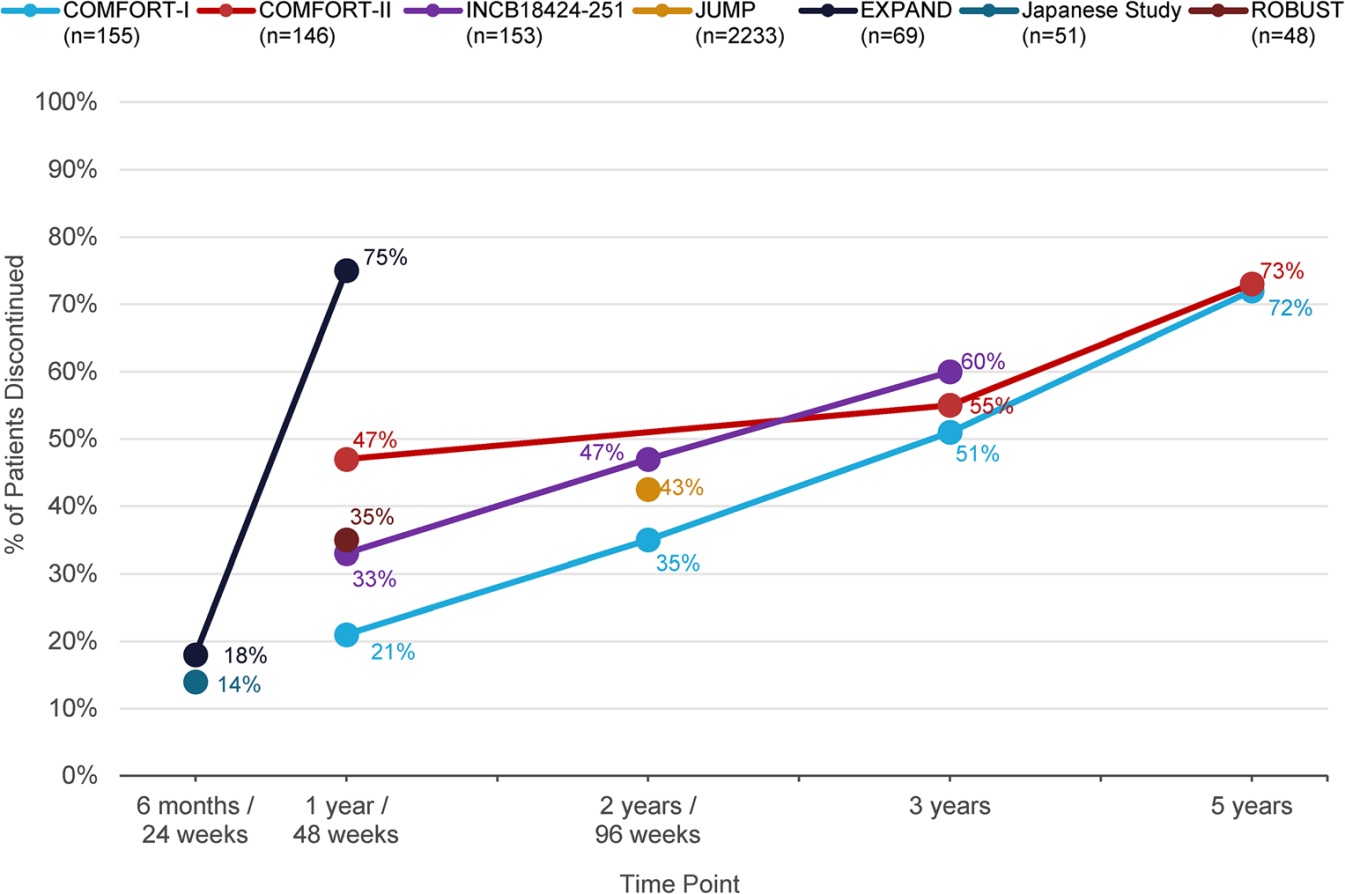
Rates of ruxolitinib treatment discontinuation at various time points in large clinical trials [23–26, 54, 55, 57, 60–62]

As noted, many patients in clinical trials permanently discontinue ruxolitinib therapy within 3 years (Fig. 2). MF symptoms and spleen size can return to pretreatment levels within approximately 1 week of discontinuing ruxolitinib [22, 24]. In some cases, ruxolitinib discontinuation may be accompanied by a “withdrawal syndrome,” attributed to rapid changes in inflammatory cytokine activity and characterized by acute onset of disease symptoms, fever, accelerated splenomegaly, worsening of cytopenias, acute respiratory distress, and occasional hemodynamic decompensation, including a septic shock–like syndrome [31, 63–66]. It is difficult to estimate the incidence of ruxolitinib withdrawal syndrome as most reports of it are in the form of cases studies. The extent of ruxolitinib exposure prior to discontinuation does not appear to influence the likelihood of developing withdrawal syndrome [31, 63–66]. However, tapering ruxolitinib doses before discontinuation and prophylactic use of glucocorticoids may reduce the likelihood of developing, or could moderate, symptoms of withdrawal syndrome [64]. When discontinuing ruxolitinib, patients should be monitored for changes in blood counts, recurring splenomegaly, and signs of respiratory distress [65]. Patients should be advised not to interrupt or discontinue ruxolitinib therapy without consulting their physician, and be informed of the potential for adverse reactions when stopping the drug [22]. If feasible, severe cases can be treated by re-initiation of ruxolitinib therapy followed by a slower taper, which may resolve withdrawal symptoms [22, 63].

### Ruxolitinib rechallenge

Data are limited as to the effectiveness of ruxolitinib rechallenge. Whether there is an efficacy difference between restarting ruxolitinib after a brief treatment interruption and that after more time has elapsed is not clear. In a preclinical study using *JAK2*-V617F leukemia cell lines subject to chronic exposure to ruxolitinib, cells that developed resistance to ruxolitinib-induced apoptosis could be resensitized to ruxolitinib after a period of ruxolitinib withdrawal [52], and case reports have suggested that ruxolitinib rechallenge can be successful in some patients. A case series included 13 patients with MF who were retreated with ruxolitinib after loss of an initial response or inadequate response to a median initial ruxolitinib duration of 62 weeks (range 6–194) [38]. All 13 patients had experienced improvement during initial treatment. Ruxolitinib rechallenge was associated with a significant spleen size reduction in 9 patients and symptom improvement in 12 patients. Four patients received a second rechallenge with ruxolitinib, and all 4 experienced some improvement in spleen length and constitutional symptoms [38]. It is unclear to what extent this is a useful strategy or how commonly it is used in routine practice.

## JAK inhibitor therapies after ruxolitinib discontinuation

The majority of patients ultimately discontinue ruxolitinib treatment and require further MF therapy [26, 29]. A challenge to second-line MF treatment is that disease biology may have evolved, making treatment responses more difficult to attain [48]. Some evidence suggests that second-line JAK inhibitor therapy may be more effective than the use of conventional treatment approaches (hydroxyurea, ESAs, danazol) after ruxolitinib failure [28, 36]. Fedratinib, pacritinib, and momelotinib can induce responses in the post-ruxolitinib setting (Table 2).

**Table:** Clinical trial data for JAK inhibitors evaluated in patients with MF previously treated with ruxolitinib [34–37]

Study	*N*	Treatment arm(s)	Control arm	Median follow-up (median study drug exposure)	Reported study drug discontinuation rate (%)	Dose modifications*	Spleen volume response^†^	MFSAF symptom response
JAKARTA2	97	FEDR 400 mg QD	N/A	6 months (24 weeks)	35^‡^	Interruption (≥ 7 days), 26% Dose reduction, 39%	55% (per protocol analysis) 31% (updated ITT analysis)	26% (per protocol analysis) 27% (updated ITT analysis)
PERSIST-2	211	PAC 400 mg QD	BAT, including RUX (45%)	NR (24 weeks)	40^‡^	20%	15% (*P* = 0.02 vs. BAT)	17% (*P* = 0.7 vs. BAT)
		PAC 200 mg BID			29^‡^	12%	22% (*P* = 0.001 vs. BAT)	32% (*P* = 0.01 vs. BAT)
SIMPLIFY-2	104	MOME 200 mg QD	BAT, including RUX (89%)	168 days (19.5 weeks)	34	16%	7% (*P* = 0.9 vs. BAT)	26% (*P* = 0.0006 vs. BAT)

*BAT* best available therapy, *BID* twice daily, *FEDR* fedratinib, *ITT* intention-to-treat, *MFSAF* Myelofibrosis Symptom Assessment Form, *MOME* momelotinib, *N/A* not applicable, *NR* not reported, *PAC* pacritinib, *QD* once daily, *RUX* ruxolitinib

*Includes dose reductions and/or treatment interruptions

^†^Proportion of patients who achieved a ≥ 35% reduction in spleen volume from baseline at week 24

^‡^Excluding discontinuations due to study holds

### Fedratinib

Fedratinib is an oral kinase inhibitor with activity against wild-type and mutationally activated *JAK2* and FMS-like tyrosine kinase 3 (*FLT3*) [32]. Fedratinib has a half-maximal enzyme inhibitory concentration (IC_50_) value for wild-type JAK2 and JAK2-V617F (3 nM) that is 35 times lower than that for JAK1, > 300 times lower than that for JAK3, and > 100 times lower than that for TYK2 [67]. Fedratinib is a more selective inhibitor of JAK2 than ruxolitinib [68] and has a longer effective half-life (~ 41 h vs. 3 h, respectively), which allows more persistent JAK2 inhibition and once-daily dosing [32, 69]. In the phase III placebo-controlled JAKARTA trial of fedratinib in JAK inhibitor–naïve patients with intermediate 2- or high-risk primary or secondary MF, the rate of spleen volume response (≥ 35% reduction from baseline spleen volume) at week 24 was 47%, which was comparable to rates in similar patients treated with ruxolitinib in the phase III COMFORT-I (42%) and COMFORT-II (32%) studies [23, 24, 39].

The phase II, single-arm JAKARTA2 trial of fedratinib in patients with intermediate- or high-risk MF previously treated with ruxolitinib (NCT01523171) was initiated in 2011, at approximately the same time that ruxolitinib was approved for treatment of MF. In 2013, the fedratinib clinical development program was placed on clinical hold by the US FDA following reports of suspected Wernicke’s encephalopathy (the clinical hold was lifted in November 2017). At the time of the clinical hold, all ongoing patients in fedratinib clinical trials, including JAKARTA2, were required to discontinue fedratinib treatment, and the studies were immediately stopped. Overall, 65% of all patients in the JAKARTA2 trial discontinued fedratinib treatment due to the clinical hold [34].

JAKARTA2 enrolled patients who were resistant to ≥ 14 days of prior ruxolitinib exposure or deemed ruxolitinib intolerant after any ruxolitinib treatment duration, per the judgment of the enrolling investigator [34]. Key inclusion criteria were age ≥ 18 years; intermediate 1- (with symptoms), intermediate 2-, or high-risk primary, post-PV, or post-ET MF; palpable splenomegaly (≥ 5 cm below the left costal margin), Eastern Cooperative Oncology Group (ECOG) performance status score ≤ 2; and platelet count ≥ 50 × 10^9^/L. Patients received initial oral fedratinib doses of 400 mg once daily in repeated 28-day treatment cycles. The primary endpoint was spleen volume response rate, the proportion of patients achieving a ≥ 35% reduction from baseline spleen volume at the end of cycle 6 (EOC6), and a key secondary endpoint was symptom response rate (≥ 50% reduction in total symptom score (TSS) on the modified Myelofibrosis Symptom Assessment Form (MFSAF) [70]).

In all, 97 patients were enrolled and treated in JAKARTA2 and comprise the intention-to-treat (ITT) population. The median age was 67 years (range 38–83). Participants generally had poor prognostic disease features at study entry: median baseline spleen volume was 2894 mL (range 737–7815), 79% of patients had received 2 or more prior MF-directed therapies, 34% had baseline platelet counts of 50 × 10^9^/L to < 100 × 10^9^/L, and 53% had baseline Hgb levels < 10 g/dL [37]. Despite the minimal exposure required to enter the trial, median prior exposure to ruxolitinib in the ITT Population was substantial, at a median of 10.7 months (range 0.1–62.4). Most patients (71%) had received ruxolitinib at initial doses of 30 mg to 40 mg daily [37].

Originally, JAKARTA2 results were reported for a “Per Protocol” subgroup of patients who had spleen volume assessments both at baseline and at least one post-baseline time point, comprising 83 (86%) of the 97 enrolled patients [34]. Analyses utilized a last-observation-carried-forward method, in which spleen volume data was “carried forward” for patients missing EOC6 assessments [34]. In the Per Protocol population, 55/83 patients (66%) were considered by enrolling investigators as resistant to ruxolitinib and 27 (33%) were deemed ruxolitinib intolerant (1 patient was classified as “other: insufficient efficacy”). The median duration of prior ruxolitinib exposure in the Per Protocol population was 10.25 months. Fedratinib was associated with an overall spleen volume response rate at EOC6 of 55% (95% CI 44%, 66%), and the symptom response rate at EOC6 was 26% [34]. Spleen volume response rate was somewhat higher in ruxolitinib-intolerant patients (63%) than in ruxolitinib-resistant patients (53%) [34]. Ruxolitinib-resistant patients were further subdivided into those with no response or stable disease during ruxolitinib treatment, those with disease progression (i.e., increased spleen size during ruxolitinib treatment), or those with a loss of response at any time during prior ruxolitinib treatment, as reported by the investigator. In these subgroups, spleen volume response rates at EOC6 were 53%, 38%, and 61%, respectively [34].

**Fig. 3:**
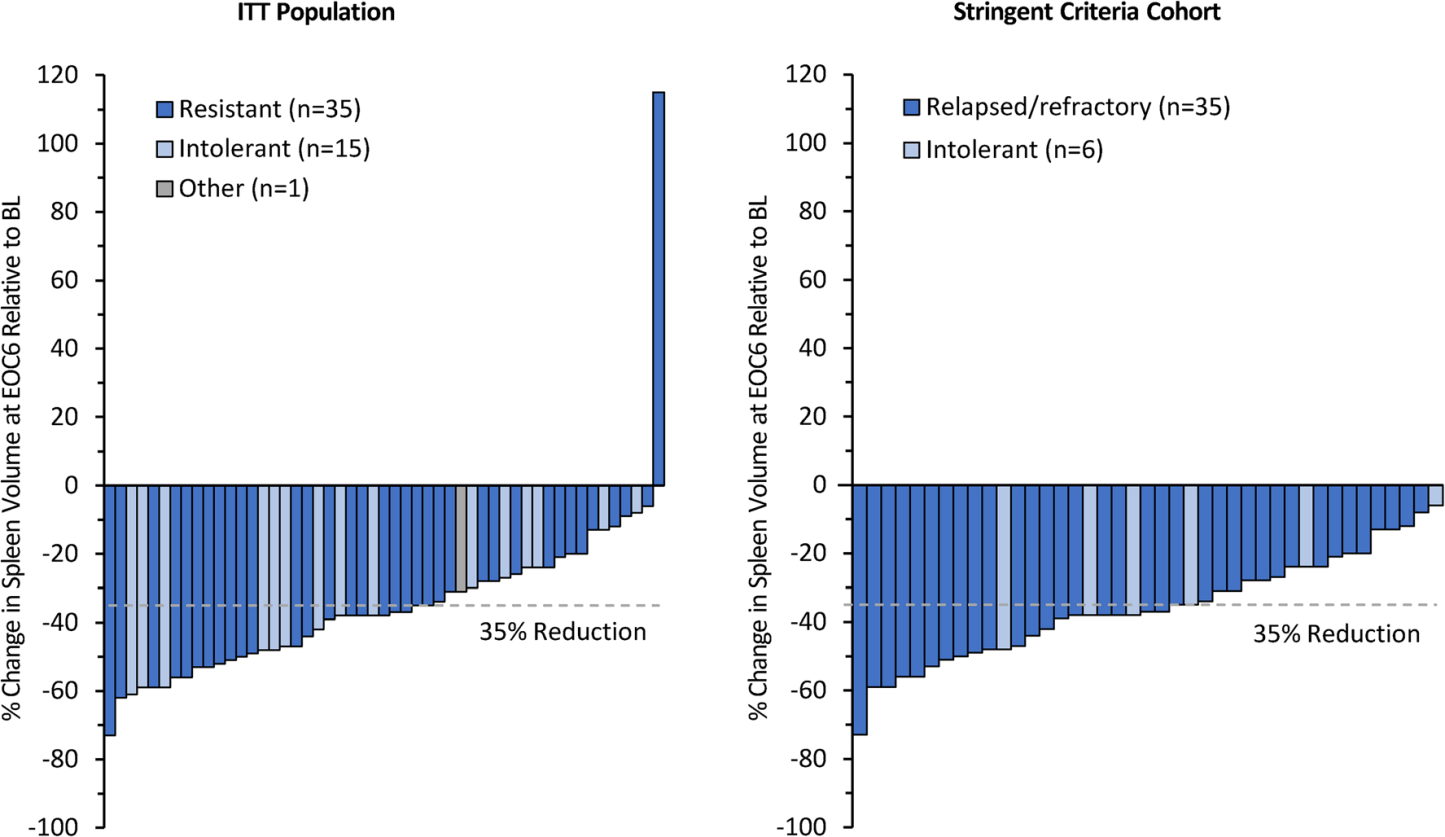
JAKARTA2. Reduction in spleen volume from baseline at the end of cycle 6 with fedratinib the ITT population (*N* = 97) and the Stringent Criteria Cohort (*N* = 97). The figure shows data assessment of patients at both time points

An updated analysis of JAKARTA2 data was recently performed to confirm the efficacy of fedratinib by employing ITT analysis principles for all 97 patients, with no imputation of missing spleen volume data [37]. In the ITT Population, the median prior ruxolitinib exposure was 10.7 months (range 0.1–64.2). With a median fedratinib treatment duration of 6 cycles (range 1–20), the spleen volume response rate in the ITT population was 31% (95% CI 22%, 41%) and the symptom response rate in the MFSAF Population (*n* = 90) was 27% [37]. All but 1 patient with baseline and EOC6 spleen volume assessments experienced some degree of spleen volume reduction during fedratinib treatment (Fig. 3). The median duration of spleen volume response was not reached (25% of 47 responding patients had a spleen response duration of < 9.4 months). Of the 47 responders in JAKARTA2, 2 patients (4%) lost response before the study was terminated [37]. Fedratinib response by prior ruxolitinib treatment outcome was also investigated [71]. Of all 97 patients, 64 (66%) were ruxolitinib resistant and 32 (33%) were ruxolitinib intolerant per investigators; median prior ruxolitinib exposures in these subgroups were 11.7 months and 7.0 months, respectively. The spleen volume response rate in ruxolitinib-resistant patients was 33% (95% CI 22%, 46%), and that in ruxolitinib-intolerant patients was 28% (14%, 47%) [71].

The updated JAKARTA2 analyses also evaluated fedratinib efficacy in a patient subgroup (*n* = 79, 81%) that met more stringent criteria for ruxolitinib relapsed, refractory, or intolerant than were used in the original analysis (Stringent Criteria Cohort) (Table 1). The median duration of prior ruxolitinib exposure before study entry in the Stringent Criteria Cohort was 11.5 months (range 1.0–62.4) [37]. The median number of fedratinib treatment cycles in the Stringent Criteria Cohort was 7 (range 1–20). Outcomes in the ITT Population were supported by the spleen volume response rate in the Stringent Criteria Cohort, which was 30% (95% CI 21%, 42%) [37], and by symptom response rate, which was the same as in the ITT Population (27%) [37]. All patients in the Stringent Criteria Cohort with baseline and EOC6 assessments experienced some degree of spleen volume reduction (Fig. 3). In this cohort, using the new stringent ruxolitinib failure criteria, 18 patients (23%) met the definition of ruxolitinib relapsed, 47 patients (59%) were ruxolitinib refractory, and 14 (18%) were ruxolitinib intolerant. Median prior ruxolitinib exposures in these groups were 11.8 months, 11.4 months, and 8.7 months, respectively. Spleen volume response rates in ruxolitinib-relapsed, refractory, and intolerant patients were similar, at 28% (95% CI 10%, 54%), 32% (19%, 47%), and 29% (8%, 58%), respectively [71].

The safety profile of fedratinib in JAKARTA2 was generally consistent with that observed in JAK inhibitor–naïve patients treated with fedratinib in other studies [39, 72]. Fedratinib dose reductions were reported for 38 patients (39%) [34]. The most common reasons for dose reductions were gastrointestinal events (16%), anemia (8%), and thrombocytopenia (6%) [34]. Most anemia and thrombocytopenia events occurred within the first four fedratinib treatment cycles [32, 72].

Fedratinib carries a black box warning for encephalopathy, including Wernicke’s encephalopathy, [32] a neurological emergency resulting from thiamine (vitamin B_1_) deficiency. Although Wernicke’s encephalopathy is commonly associated with a history of alcohol abuse, it is also observed in patients with other malnourished states due to malabsorption, poor dietary intake, or increased metabolic requirement [73, 74]. No Wernicke’s encephalopathy occurred in the JAKARTA2 study, although 1 patient developed hepatic encephalopathy [37]. Thiamine levels and nutritional status should be assessed in all patients before starting fedratinib, periodically during treatment, and as clinically indicated [32, 44].

Currently, the most extensive exposure to fedratinib therapy occurred in the extension portion of a phase I dose-finding and expansion study in adult patients with JAK inhibitor–naïve MF (NCT00631462, NCT00724334) [75, 76]. In an interim analysis from that study, 23 patients had received fedratinib treatment for a median of 30 cycles (range 13–44) at a median daily dose of 440 mg [76]. The proportion of patients with a ≥ 50% reduction in spleen size from baseline was 61% at 30 months (*n* = 18), and no unexpected safety signals emerged during long-term fedratinib therapy [76]. Long-term outcomes of fedratinib treatment in patients previously treated with ruxolitinib are currently under investigation. The open-label, single-arm, multicenter, phase IIIb FREEDOM study (NCT03755518) and the randomized, multicenter, open-label, phase III FREEDOM2 study of fedratinib versus BAT (NCT03952039) are ongoing to evaluate long-term clinical response and safety, survival outcomes, and risk mitigation strategies for managing gastrointestinal adverse events and Wernicke’s encephalopathy during fedratinib therapy.

### Pacritinib and momelotinib in patients previously treated with ruxolitinib

Pacritinib, like fedratinib, is a JAK2/FLT3 inhibitor [77]. The phase III PERSIST-1 study enrolled patients with JAK inhibitor–naïve MF [41]. The phase III PERSIST-2 trial of pacritinib (200 mg BID, *n* = 74; 400 mg QD, *n* = 75) versus BAT (*n* = 72) (NCT02055781) enrolled patients with intermediate- or high-risk MF and platelet counts ≤ 100 × 10^9^/L, including patients who had received prior treatment with “1 or 2 JAK inhibitors” [36]. Previous ruxolitinib exposure and outcomes of prior ruxolitinib treatment for patients in PERSIST-2 have not been described [36]. The spleen volume response rate for all pacritinib-treated patients in PERSIST-2 (regardless of prior JAK inhibitor exposure) was 18%, and the symptom response rate was 25% [36]. A total of 62 patients (42%) in the two pacritinib treatment arms had received prior ruxolitinib therapy; in these patients, pacritinib (both doses combined) induced a spleen response rate of 10% and a symptom response rate of 21% [36]. For patients with baseline platelet counts < 50 × 10^9^/L, there was no evidence of increasing thrombocytopenia in the pacritinib or BAT arms during treatment. Concern over high-grade cardiac and hemorrhagic events in the PERSIST studies led to implementation of the PAC203 trial, a dose-finding study of pacritinib in patients for whom ruxolitinib had failed (NCT03165734), which evaluated the efficacy of the 200 mg BID dose assessed in PERSIST-2 study, as well as lower pacritinib doses (100 mg QD and 100 mg BID) [78]. Patients must have been intolerant to ≥ 28 days of ruxolitinib exposure (developed RBC transfusion dependence, or grade ≥ 3 anemia, thrombocytopenia, or hemorrhage while receiving ≤ 20 mg BID ruxolitinib) or failed to benefit from ruxolitinib treatment after ≥ 3 months (< 10% spleen volume reduction or < 30% decrease in spleen length or regrowth of these parameters). Results of the PAC203 trial showed greatest spleen volume reductions and TSS improvement with the pacritinib 200 mg BID dose [78]. Approximately one fourth of all MF patients become thrombocytopenic within 1 year from diagnosis [79]; pacritinib may be a good initial therapeutic option for patients with MF who present with severe thrombocytopenia [80].

In addition to spleen and symptom improvements, momelotinib may especially benefit patients with significant MF-associated anemia [35]. Momelotinib is a JAK1/JAK2 inhibitor, which in murine models of anemia in chronic disease, was shown to inhibit bone morphogenic protein receptor kinase activin A receptor type I (ACVR1)–mediated hepcidin expression, which stimulated erythropoiesis [81]. In the phase III SIMPLIFY-2 study of momelotinib versus BAT in patients with intermediate- or high-risk MF (NCT02101268), eligibility criteria required prior or current exposure to ruxolitinib for ≥ 28 days, with either a need for RBC transfusions while on ruxolitinib, or of a dose adjustment of ruxolitinib to < 20 mg BID with either grade 3 thrombocytopenia or anemia, or bleeding at grade ≥ 3 during ruxolitinib treatment [35]. A potential disadvantage of the eligibility criteria in SIMPLIFY-2 is that it excluded patients who may have tolerated but were refractory to ruxolitinib treatment [33, 35]. It is worth noting that patients could have had stable disease or a spleen response during prior or current ruxolitinib therapy at study entry (but with suboptimal hematologic response or toxic effects) and there was not a prespecified ruxolitinib “washout” period before study entry [35]. In SIMPLIFY-2, momelotinib was not superior to BAT for inducing a ≥ 35% reduction in spleen volume at week 24 (7% vs. 6%, respectively); however, 89% of patients in the BAT arm were receiving ruxolitinib on-study [35]. In secondary endpoint analyses, more patients in the momelotinib arm were transfusion independent at week 24 than patients in the BAT arm (43% vs. 21%, nominal *P* = 0.0012), and 40% of momelotinib-treated patients required no transfusions over the treatment phase, compared with 27% of patients in the BAT group (nominal *P* = 0.10) [35]. The effects of momelotinib on stimulating erythropoiesis, inducing anemia responses, and reducing transfusion burden [82] suggest patients with significant MF-associated anemia may especially benefit from the drug.

## Summary/conclusions

Ruxolitinib is the JAK inhibitor drug with the most mature data and longest follow-up for efficacy and safety in patients with MF. While ruxolitinib induces spleen volume reductions and improves symptoms in most treated patients, many patients discontinue the drug within a few years. Ruxolitinib rechallenge may be effective for inducing responses in a small number of patients. Until the approval of fedratinib in 2019, no alternative JAK inhibitor was available for second-line MF treatment in cases of ruxolitinib failure. The National Comprehensive Care Network clinical practice guidelines for myeloproliferative neoplasms now recommend fedratinib as initial treatment, and as second-line therapy if there is no response or loss of response to ruxolitinib [44]. In JAKARTA2, fedratinib showed robust efficacy and a manageable safety profile in the post-ruxolitinib setting in patients with poor prognostic MF features at study entry, including patients who met new stringent definitions of prior ruxolitinib failure [37]. Nevertheless, due to the temporary clinical hold on fedratinib clinical development, long-term exposure to fedratinib is limited at this time and rates of potential secondary resistance to fedratinib remain to be determined. Pacritinib and momelotinib have shown less robust spleen responses than fedratinib in patients previously treated with ruxolitinib in clinical trials [34–36], but these drugs may have utility as second-line therapy in select patients with more severe cytopenias.

Defining ruxolitinib failure remains a matter of clinical judgment. Whether, when, and how best to initiate second-line JAK inhibitor therapy remains to be determined, but such information should become more readily available now that there is an approved alternative treatment option with fedratinib for patients with MF.

The complexity and heterogeneity of MF disease biology has prompted investigation of therapies with a variety of pathogenic targets in studies that include patients previously treated with ruxolitinib (Table 3) [83]. Increasing understanding of MF pathobiology is leading to exploration of various JAK2 inhibitor combination strategies, and of monotherapies, including epigenetic modifiers and immune regulators, that influence pathways other than JAK/STAT signaling.

**Table 3:** Examples of studies in patients with MF that require or allow previous exposure to ruxolitinib (adapted from [83])

Drug class	Drug	Mechanism of action	Clinical development phase	ClinicalTrials.gov number
JAK inhibitors	Itacitinib (± ruxolitinib)	JAK1 inhibitor	2	NCT03144687
	NS-018	JAK2 inhibitor	1/2	NCT01423851
	LY2784544	JAK2 inhibitor	2	NCT01594723
Epigenetic modifiers	Pracinostat + ruxolitinib	HDAC inhibitor	2	NCT02267278
	Panobinostat + ruxolitinib	HDAC inhibitor	1	NCT01693601
	Azacitidine + ruxolitinib	HMA	2	NCT01787487
	SGI-110	HMA	2	NCT03075826
	IMG-7289	LSD1 inhibitor	2	NCT03136185
PI3K/AKT/mTOR pathway inhibitors	Parsaclisib + ruxolitinib	PI3K inhibitor	2	NCT02718300
	Buparlisib	PI3K inhibitor	1	NCT01730248
	TGR-1202 + ruxolitinib	PI3Kδ inhibitor	1	NCT02493530
Small molecule inhibitors	CPI-0610 (± ruxolitinib)	BET inhibitor	1/2	NCT02158858
	PIM447 + ruxolitinib	Pan-PIM kinase inhibitor	1	NCT02370706
	Ribociclib + ruxolitinib	CDK4/6	1	NCT02370706
	Navitoclax (± ruxolitinib)	BCL-2 inhibitor	2	NCT03222609
	Alisertib	Aurora kinase A	Not applicable	NCT02530619
Checkpoint inhibitors	Pembrolizumab	PD-1 inhibitor	2	NCT03065400
	Nivolumab	PD-1 inhibitor	2	NCT02421354
Novel agents	Imetelstat	Telomerase inhibitor	2	NCT02426086
	Glasdegib	Hedgehog inhibitor	2	NCT02226172
	PRM-151	Pentraxin 2 agonist	2	NCT01981850
	Sotatercept	TGF-β ligand trap	2	NCT01712308
	P1101	Peg-interferon-α	2	NCT02370329
	LCL-161	Mitochondrial-derived activator of caspases (SMAC) mimetic	2	NCT02098161

*BCL-2* B-cell lymphoma 2, *BET* bromodomain and extraterminal domain, *HDAC* histone deacetylase, *HMA* hypomethylating agent, *JAK*, Janus kinase, *LSD1* lysine-specific demethylase 1, *mTOR* mammalian target of rapamycin, *PI3K* phosphoinositide 3-kinase, *PD-1* programmed cell death protein 1, *TGF-β* transforming growth factor beta
